# Laser speckle size and contrast investigation of volumetric scattering from controlled turbid phantoms and mouse skin tissues

**DOI:** 10.1016/j.isci.2025.112433

**Published:** 2025-04-15

**Authors:** Carla Kulcsar, Daniel C. Louie, Alex Vitkin

**Affiliations:** 1 Department of Medical Biophysics, University of Toronto, Toronto, ON M5G 1L7, Canada; 2 Princess Margaret Cancer Centre, University Health Network, Toronto, ON M5G 1L7, Canada; 3 Department of Radiation Oncology, University of Toronto, Toronto, ON M5T 1P5, Canada

**Keywords:** Optics, Photonics, Biomedical engineering

## Abstract

The potential of spatial laser speckle analysis to interrogate scattering properties of volumetric optical tissue-like phantoms and biological tissues is examined. The simple and inexpensive experimental setup consists of a HeNe laser illuminating turbid samples to acquire speckle patterns in backscattering geometry. Theoretical Monte Carlo simulations of subsurface light fluence patterns and scattering statistics are used to interpret the experimental findings, in an effort to help expand established speckle theory from rough surface effects to volumetric scattering processes. Beyond controlled phantom studies, initial results from normal and pathologic biological tissues are also presented and discussed. Building on previous research that attempts to link specific stochastic speckle metrics to medium scattering properties, this study suggests that a combination of speckle contrast and speckle size can be related to varying scattering coefficients, toward distinguishing samples by their underlying scatterer size and concentration.

## Highlights

• Laser speckle size and contrast report on scattering properties of turbid phantoms
• Combined usage of these metrics separates scatterer size and concentration effects
• Monte Carlo simulations help interpret volumetric speckle pattern statistics
• Speckle size and contrast show difference in normal and melanoma mouse skin tissue

## Graphical abstract

**Figure:**
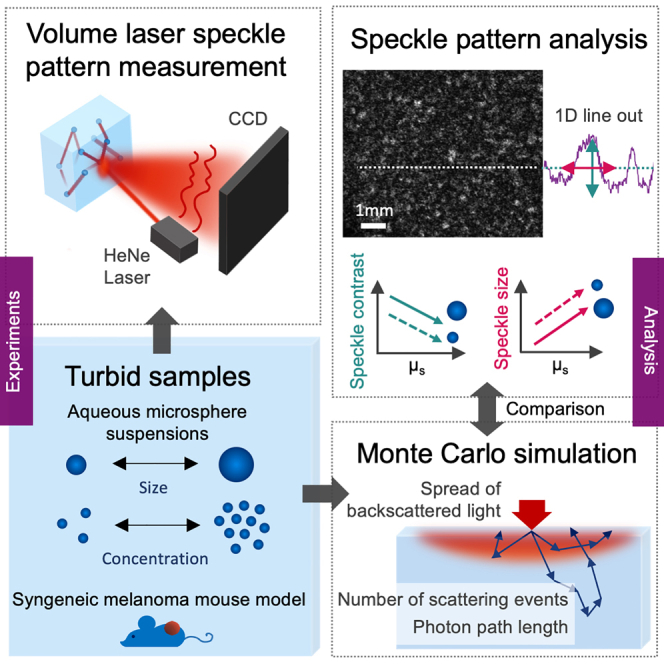

## Introduction

Noninvasive sensing of pathology-related changes in optical scattering properties of bulk tissue remains a challenge in today’s biophotonics. Methods like optical coherence tomography,^1^ diffuse reflectance spectroscopy,^2^ spatial frequency domain imaging,^3^^,^^4^^,^^5^ and integrating sphere measurements^6^ have been developed to estimate the optical properties of tissues, but these often assume some *a priori* knowledge for reconstruction^7^ and have difficulty in measuring bulk tissues.

As an inexpensive simple-instrumentation approach that can be conducted in a backscattering geometry suitable for bulk tissue measurements, laser speckle analysis offers a potential route to investigate the optical properties of tissues. Speckles arise from the interference of coherent light waves interacting with a scattering volume, imposing phase differences on the incident wavefront and causing constructive/destructive interference when the illuminated medium (tissue) is imaged. The resultant variations in detected image intensity thus represent an interference pattern arising from sub-resolution scatterers in the sample. It may be possible to relate the resultant speckle characteristics to underlying medium properties such as the scattering properties of bulk biological tissues.

Laser speckle analysis has found many applications in preclinical and clinical biomedical optics,^8^^,^^9^^,^^10^ primarily in imaging of dynamic processes.^11^ One important example is the temporal decorrelation of speckle patterns for imaging blood flow.^11^^,^^12^ While *time-varying* speckles depend on the motion of optical scatterers in a sample,^13^ *spatial* analysis of stationary speckle patterns has also been used to explore the underlying bulk medium properties such as the scatterer size, concentration, and composition. This may prove useful in the diagnosis of cancerous tissue as pathological processes alter these tissue scattering properties. Alterations in tissue morphology and composition during cancer progression that influence its optical properties are manyfold, with relevant biomarkers ranging from nuclear pleomorphism in cellular compartments^14^ to variations in collagen fiber and water concentration in the stroma.^15^^,^^16^^,^^17^^,^^18^ These may lead to detectable differences between speckle patterns acquired from healthy and cancerous tissue, as explored in the following.

The examination of stochastic properties of a spatially varying speckle pattern may thus provide insights into the optical properties of the interrogated sample the speckles arise from. For quantification, speckle size and speckle contrast are often the selected metrics as they (1) are directly calculable and (2) have some theoretical underpinnings relating them to medium properties and/or measurement geometry. However, despite their relative measurement simplicity, speckle patterns from complex turbid heterogeneous media such as biological tissues are too difficult to understand and interpret, offering limited insight into the underlying scattering interactions and medium properties of interest. Thus, systematic experiments in controlled optical phantoms such as uniformly sized microsphere suspensions are more likely to furnish unambiguous interpretation of speckle characteristics-medium properties links, revealing useful dependencies and trends toward complex biological tissue examinations. For example, previous work has shown that speckle size is affected by the size of the scatterers^19^ as well as the proportion of large and small scattering microspheres in a mixture^20^ in different scattering regimes. Furthermore, it has been shown that speckle size tends to increase with increasing scattering coefficient^21^ and is sensitive to the scattering anisotropy factor g^22^ when measured in backscattering geometry. These empirical trends have been explained qualitatively by relating them to the change of the spot size of the light exiting the scattering medium due to its varying optical confinements caused by different sample turbidities.

While these results are promising in understanding the generation of speckle patterns, more speckle metrics are needed to more directly and unambiguously link the image characteristics to underlying medium properties. For example, Piederriere et al. note that trends of speckle size (measured in transmission mode) for varying optical thicknesses overlap for certain sphere sizes; they then suggest an additional metric of speckle contrast to differentiate the resultant overlapping curves.^23^ Speckle contrast metric has previously shown promising results in exploring surface roughness in biomedical applications,^24^ and Goodman et al. have developed an extensive theory on the generation of speckle patterns from rough surfaces.^25^ However, a quantitative connection between volumetric scattering properties and speckle contrast remains to be explored.

In this study, we advance previous work by quantitatively investigating speckle size and speckle contrast generated from turbid volumetric samples containing uniform-sized microspheres. Our analysis supplements experimental measurements with optical Monte Carlo (MC) simulations of photon subsurface fluence profiles and pathlength-related quantities, facilitating speckle results interpretation and enabling comparison of theoretical formalisms proposed in the literature^25^ to volumetric scattering properties. This work adds to the preceding studies by demonstrating that speckle theory based solely on surface analysis is insufficient for describing volume speckle formation, pointing to further need for relating laser speckle pattern statistics to sample volumetric scattering characteristics. Furthermore, while selected prior studies had focused on trends in speckle size and speckle contrast separately, it remains unclear how these can be used in quantitative interpretations of biomedical speckle patterns. Here, we demonstrate that an innovative parametric combination of both speckle size and contrast metrics may resolve scatterer size and concentration in turbid media approximating biological tissues; preclinical proof of principle is also demonstrated in a syngeneic melanoma mouse model. From these findings, potential exploration avenues to build on this initial study are suggested in the conclusion.

## Results and discussion

### Polystyrene microsphere phantoms

**Figure 1:**
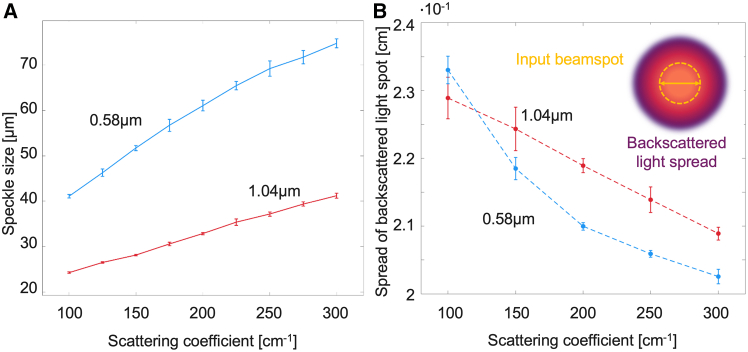
Speckle size analysis and Monte Carlo simulated spread of backscattered light (A) Experimental results for speckle size (calculated through the full-width half maximum of the autocorrelation function) as a function of the increasing scattering coefficient. A linear increase is visible for both sphere sizes. (B) Full-width half maximum of light intensity emitted from the front surface of the sample obtained by Monte Carlo simulation for input beam diameter of 2 mm. The light appears more confined for smaller spheres and decreases for both sizes with increasing turbidity. In both (A) and (B), lines are a guide for the eye. Data are represented as mean +/− standard deviation.

Figure 1A shows the speckle size as a function of increasing turbidity for the two microsphere diameters. A high scattering coefficients range was chosen to simulate tissue scattering properties.^26^^,^^27^ As seen, speckle size increases linearly with an increasing scattering coefficient, and smaller-sized microspheres exhibit larger speckles. Such increasing speckle size with turbidity has previously been observed.^21^^,^^28^ To explain this behavior, note that, as the medium scattering increases, the retro-reflected light is less spread out relative to the incident illumination spot size (∼2 mm diameter in our case), such that D_l_ term in Equation 1 decreases. Due to its inverse relationship with speckle size as per that equation, the increasing trends of the two curves in Figure 1A are observed. Further, for a given scattering coefficient, this spot confinement is expected to be greater for smaller scatterers owing to differences in scattering anisotropy g (the phase functions for smaller scatterers has a greater probability for large-angle backscattering compared to larger microspheres, thus greater light confinement). Thus larger speckles for smaller microspheres are expected, as in indeed borne out in Figure 1A.

To explore these light confinement-speckle size mechanisms further, Figure 1B presents MC simulation results of the profiles of the backward-exiting light intensity. For these highly turbid samples, a 2-mm-diameter incident beam spot manifests full-width half maxima (FWHM) of the exiting profile with dimensions of ∼2.3 (smaller scattering coefficients) to ∼2.05 mm (larger scattering coefficients). Thus the D_l_ term in Equation 1 indeed decreases, leading to larger speckles with increasing turbidity. The fact that the light spread decreases more steeply for smaller spheres is reflected in the experimental findings of a larger speckle size-turbidity slope for smaller spheres. Further, this light spread confinement effect is in general greater for smaller spheres (at a given turbidity level), again in accord with experimental findings of larger speckles for smaller scatterers seen in Figure 1A. But, while these two qualitative agreements (turbidity and size dependencies) between experiment and theory are encouraging, quantitative agreement is less so. That is, the MC-predicted decrease in D_l_ (for the range of examined turbidities) is not commensurate with the correspondingly larger increase in speckle size seen experimentally (∼1.8X). Further, the MC-predicted modest difference in light confinement for the two microsphere sizes is likewise smaller than the corresponding experimentally observed speckle size differences, especially at low scattering coefficients, where the MC light spreads even overlap. Such qualitative-only agreements suggest that perhaps the underlying theory represented by Equation 1 is incomplete for volumetric scattering scenarios. For example, the influence of scattering anisotropy g may play an important role and appear explicitly in the volume-scattering analog of Equation 1, over and above the light spot size exiting the surface of the sample currently being the only medium-dependent characteristic.

**Figure 2:**
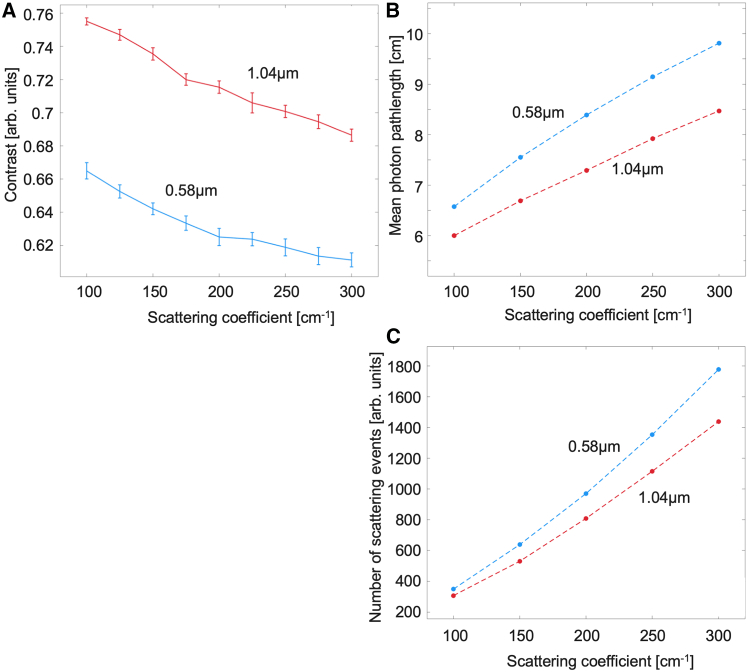
Speckle contrast analysis and Monte Carlo simulated mean photon pathlength and number of scattering events (A) Experimental results from microsphere suspension for speckle contrast, seen to decrease with decreasing turbidity and decreasing scatterer size. Monte Carlo simulation results for (B) mean total pathlength and (C) mean number of scattering events for different scattering coefficients and scatterer sizes. Lines on all plots are a guide for the eye. Data are represented as mean +/− standard deviation.

Figure 2A shows the other calculated speckle pattern metric, that of speckle contrast. Unlike speckle size, speckle contrast decreases with turbidity and is larger for larger spheres. This behavior is in accord with the general observation of contrast being higher in less-scattering media.^23^^,^^29^ The contrast being higher for larger spheres also agrees with previous transmission-geometry observations.^23^ Although transmission polarimetric measurements through thin samples often do not show the same trends as reflection-mode results from corresponding thick bulk samples,^22^ it is interesting to note here that the dependency of speckle contrast on scatterer size and medium turbidity seems consistent in both transmission and backscattering measurement geometries.

While the theory relating speckle contrast to medium’s surface roughness properties has been established,^25^^,^^30^ the linkages to bulk (volumetric) tissue properties have not been thoroughly investigated. To gain some insights, MC simulation results (Figures 2B and 2C) show the calculated estimates of the total number of scattering events and total photon pathlengths. While the total pathlength and number of scattering events are closely connected—their ratio is the mean free path—they represent slightly different physical mechanisms happening to the propagating coherent light. Specifically, Goodman and others^25^^,^^31^ mention random pathlengths as the main driving force behind speckle pattern generation in volume scattering by introducing phase delays into the wavefront, resulting in interference-generating phase differences when the light exits the medium. Further, the total number of scattering events may possibly be linked to the number of independent phasors invoked in basic speckle theories^25^ as it reflects on the number of times the phase of the light is changed at each scattering event.

As shown in Figure 2B, the total pathlength increases with turbidity. As the light loses coherence with increasing total pathlength through the medium, fringe visibility in the resulting interference pattern decreases. Thus the measured speckle contrast—a measure of this visibility—decreases anti-proportional to the total pathlength. The loss in coherence by scattering through the medium may indicate that the resulting contrast is dependent on the coherence length of the incident illumination source. Specifically, we expect increased contrast dynamic range when the coherence length of the light source is on the order of the total pathlength distribution of the scattered photons.^32^^,^^33^ In the experiments presented here, the HeNe laser has a coherence length greater than 20 cm and, while perhaps not optimal, ensures that the scattered light interferes and creates observable speckle patterns with adequate dynamic range to sense scattering differences. A source with a shorter coherence length (of the order of several cm, to match the ordinate axis in Figure 2B) might increase the dynamic range of speckle contrast for varying-turbidity samples. Indeed, surface roughness speckle pattern studies improve when using laser diodes sources, due to their mm-scale coherence lengths better matching the rough surface height variations.^24^^,^^34^ The analytical models used to explore this have not yet advanced to encompass volumetric scattering pathlengths.

The increase in the number of scattering events, as shown in Figure 2C, supports our supposition that the number of total pathlength/number of scattering events is linked to the theoretical value of “independent phasors” N, established for surface speckle theory but not explored so far in volumetric measurements. We posit that relative changes in speckle contrast due to an increase in the number of scattering events (or number of independent phasors) occur regardless of the coherence length of the laser source and can therefore be interpreted as a measure of medium characteristics for arbitrary illumination spectral profiles.

**Figure 3:**
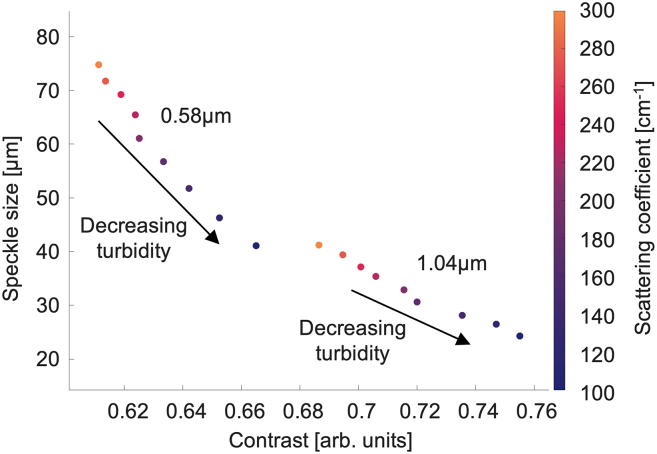
Combination of speckle size and contrast in optical phantom imaging Combined plot of speckle size and contrast for each experimentally measured scattering sample. The scattering coefficients are represented by a color bar on the right (blue to orange ranging from μ_s_ = 100 to 300 cm^−1^).

Since the actual values of both speckle size and contrast are likely dependent not only on medium properties but also on measurement details (detection angle, magnification, etc), one can work with *relative* changes in these metrics as presented in this paper so far. To further minimize the role of measurements details, we focus on speckle characteristics that are dependent primarily on medium properties and thus investigate *combined* speckle size and contrast analysis to distinctly differentiate volumetric samples based on their scattering properties. Figure 3 plots the two (independent^35^) speckle metrics against each other, yielding two separate clusters for the two examined scatterer sizes. The resulting separation in the speckle-metrics parameter space based on scatterer size is encouraging in the context of speckle-based medium characterization. Further, the dependence on medium turbidity is also evident, and the slope of this dependence is (slightly) different for the two groups. Since the slope of the curves appears to depend on the sphere size, it could potentially serve as another parameter for differentiating samples. Also, the sphere-size-dependent scattering anisotropy is a physical quantity affecting both the spot size and sampling volume, and we will investigate in a future study if the slope of this cluster plot is influenced by the scattering g-factor.

Conceptually, Figure 3 can be thought of as a first-pass “look-up table” for determining sample properties from speckle measurements. For example, small speckles and large contrast suggest a medium with lower concentration of large scatterers; conversely, larger lower-contrast speckles suggest a medium consisting of small scatterers at higher concentrations. Clearly much additional research is needed to solidify the aforementioned trends and transform Figure 3 into a proper quantifiable look-up table. For example, one obvious drawback now is the rather limited dynamic range of the speckle contrast metric (abscissa axis in Figure 3), so experiments must be optimized to focus on this metric if it is to be useful in realistic and complex cases (for example, using a better-matching coherence length source as discussed earlier). Other avenues of further research that build on this initial study of volumetric speckle effects are suggested at the end of the manuscript.

### Demonstration of speckle pattern measurement and analysis methodology in biological tissue

**Figure 4:**
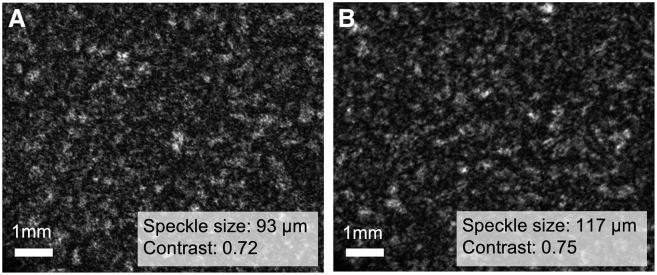
Biomedical feasibility demonstration (A) Speckle pattern acquired in backscattering geometry from mouse skin tissue adjacent to the melanoma tumor site; (B) measurement repeated on melanoma tumor site. Note the difference in calculated speckle metrics in the normal-to-pathology transition, especially in the effective speckle diameter; for details, see text. The scale bar represents 1 mm.

As proof-of-principle demonstration for potential speckle image analysis in the biological context of cancer detection and assessment, we provide data from a syngeneic mouse model containing a melanoma tumor. Figure 4 presents example images from tumor tissue and healthy bare skin adjacent to the tumor, acquired immediately *postmortem*. Note the marked difference in the calculated speckle size (effective speckle diameter): 93 ± 3 μm in normal mouse skin and 117 ± 3 μm in the melanoma tumor. The contrast difference of the bulk tissue speckle patterns is more modest, 0.72 ± 0.02 for healthy tissue and 0.75 ± 0.01 for cancerous tissue. In accord with our reported phantom results, the prominent normal-to-pathology reduction in speckle size may imply an increase in scattering coefficient or a decrease in effective scatterer size. This is consistent with previous literature reports on the optical properties of melanoma, which show that the reduced scattering coefficient increases in melanoma compared to healthy skin.^36^^,^^37^ The magnitude of the observed reduction in contrast (0.72–0.75) is more modest compared to speckle size changes; in this limited proof-of-principle experiment, it may be too premature to infer contrast trends at this stage. Given that melanomas have a higher absorption coefficient than healthy skin tissue,^38^^,^^39^^,^^40^ we would expect increased contrast due to the shorter pathlengths of backscattered photons, as well as larger speckles resulting from a reduced light spot of the outgoing light. This is indeed reflected in our issue speckle measurements, but more prominently in the latter metric. Overall, it is also interesting to note that, in comparing biological speckle patterns to microsphere phantom results, larger speckles may suggest smaller scatterers in biological tissue. Again, this is consistent with tissue studies that report an average effective scatterer size on the order of small structures/subcellular organelles in mammalian cells.^41^

We are cautious in not over-interpreting this biological feasibility experiment, as there are many other biological factors at play and thus many other differences between phantoms and real tissues; for example, substantive differences in the nature of the refractive index fluctuations in the two systems—discrete 2-phase variations (phantoms) versus random continua (tissue)—must be borne in mind. Nevertheless, this initial analysis of volumetric tissue speckle images shows utility of the methodology and some promise for distinguishing normal from pathological conditions.

### Conclusion and outlook

The potential of speckle size and speckle contrast in volumetric scattering to report on medium properties was investigated through experimental measurements and MC simulations. Key dependencies of these speckle metrics on medium properties were reported under controlled experimental conditions in bulk turbid phantoms. In summary, for a given scatterer, increasing turbidity leads to an increase in speckle size and decrease in speckle contrast. At a constant turbidity, larger scatterers yield smaller speckle size and higher speckle contrast. MC simulations helped facilitate measurement results interpretation. The potentially predictive nature of combined speckle size and speckle contrast in the investigation of medium scattering properties was demonstrated. A biological proof-of-principle demonstration of volumetric speckle pattern analysis for melanoma detection in a syngeneic mouse model was provided, showing clear differences in normal vs. cancerous tissue images. Clearly much remains to be investigated in the parameter space of bulk tissue characterization with speckle measurements; this paper presents a first step toward a detailed methodology of volumetric speckle formation.

To build on this initial study, the following deeper dives into volumetric speckle generation are planned, including (1) exploring effects of the scattering anisotropy (g-factor), for example, its influence on the slope of the speckle size versus contrast curves; (2) optimizing experimental measurements to increase the contrast dynamic range, for example, by using different coherence lengths sources; (3) investigating additional independent speckle metrics beyond size and contrast (speckle density, intensity distribution-related metrics such as kurtosis, power spectral density analysis, texture analysis, and so on) to increase dimensionality of the data toward improved medium characterization (with potential for radiomics and/or AI-like analysis as data density increases); (4) measuring complex optical phantoms including mixed poly-sized spheres, asymmetric scatterers like cylinders (to simulate collagen or muscle fibers) or disks (to simulate scattering from red blood cells), and medium absorption; (5) exploring polarization effects in volumetric speckle, as polarization is significantly under-explored dimension in speckle studies that warrants an extensive investigation; and (6) adapting an MC algorithm proposed in previous literature^41^^,^^42^^,^^43^ for accurate volumetric scattering speckle pattern simulation. Overall, it is hoped that building upon the results of this initial study, these (and other) research avenues of speckle patterns from bulk turbid biological media will be of interest to, and pursued by, the biomedical optics community.

### Limitations of the study

Despite providing insights into how scattering properties of volumetric samples affect laser speckle statistics, the main limitation of this study are (1) the absence of a theoretical model—such as an MC rendered speckle pattern or a mathematical prediction—to a directly compare our experimental results to; (2) the limited dynamic range of the measured contrast metric; (3) non-usage of many additional and potentially informative higher-order speckle metrics (beyond size and contrast); and (4) the uncertain nature of the relationship between phantom and tissue results. The “deeper dives” paragraph in the conclusion briefly expands on these and other issues and proposes some initial mitigation strategies.

## Resource availability

### Lead contact

Requests for further information and resources should be directed to and will be fulfilled by the lead contact, Carla Kulcsar (carla.kulcsar@mail.utoronto.ca).

### Materials availability

This study did not generate any new unique reagents.

### Data and code availability

• Data reported in this paper can be shared by the lead contact upon reasonable request.
• All original code is available in this paper’s supplemental information (see Data S1).
• Any additional information required to reanalyze the data reported in this paper is available from the lead contact upon request.

## Acknowledgments

The authors thank Dr. Carla Calcada (Princess Margaret Cancer Research Institute, Toronto) for providing the mouse model and assisting in the animal experiments. The authors also thank University of Toronto colleagues Michael Singh and Kseniia Tumanova for productive discussions and artistic figure advice. C.K. acknowledges generous graduate studies support by the German Academic Scholarship Foundation and the OSOTF Cunningham Award. Following are the funding sources: New Frontiers in Research Fund (NFRFE-2019-01049), 10.13039/501100000024Canadian Institutes of Health Research (PJT-156110), and 10.13039/501100000038Natural Sciences and Engineering Research Council of Canada (RGPIN-2018-04930).

## Author contributions

C.K. performed the experiments and data analysis and wrote the manuscript. A.V. and D.C.L. offered scientific discussions and project guidance. A.V. provided funding support and laboratory equipment. All authors reviewed and edited the manuscript.

## Declaration of interests

The authors declare no competing interests.

## STAR★Methods

### Key resources table

**Table:** 

REAGENT or RESOURCE	SOURCE	IDENTIFIER
**Experimental models: Cell lines**		

S91 cell line	ATCC	Ccl 53.1

**Experimental models: Organisms/strains**		

DBA mice	Jackson laboratory	JAX:000671

**Software and algorithms**		

Polarization sensitive Monte Carlo engine	Côté et al.^44^	http://www.novajo.ca/ont-canc-inst-biophotonics
MATLAB version 2023b	MATLAB - MathWorks	https://www.mathworks.com
Code for analyzing speckle size and speckle contrast of raw speckle images	This paper	N/A

**Other**		

Polystyrene Microspheres	Bangs Laboratories, Inc	LOT#12487, LOT#5701

### Experimental model and study participant details

#### Syngeneic melanoma mouse model

For the proof-of-principle, we imaged cancerous vs. healthy tissue in *n* = 1 mouse. An immunocompetent male DBA mouse was intradermally inoculated with mouse melanoma cancer cells (S91 cell line, gender: male) at 6 weeks of age. The tumor, located on the flank of the mouse, grew for 2 months, and upon reaching a diameter of ∼3.5mm, the mouse was sacrificed for subsequent immunohistochemical analysis. The tissue speckle imaging was performed immediately after sacrifice. The positioning of the healthy and tumor tissue sites relative to the measurement setup remained consistent to minimize system effects on the speckle metrics. All animal procedures were performed in accordance with the Guide to the Care and Use of Experimental Animals set forth by the Canadian Council on Animal Care. Experiments were performed according to a protocol approved by the University Health Network Institutional Animal Care and Use Committee in Toronto, Canada.

### Method details

#### Optical phantoms

To achieve optical phantoms with controlled and variable optical scattering coefficients, polystyrene microspheres (Bangs Laboratories, Inc) were suspended in water. These offer precise user-defined and calculable control over optical properties and a reliably reproducible protocol without complexities Optical Phantomsassociated with static optical phantoms. Emulating malignancy-related changes in tissue optical properties, for example variations in nuclear size and changes in turbidity,^45^^,^^46^^,^^47^ we investigate speckle pattern images for a range of scattering coefficients (100-300cm^−1^) for two different sizes of spheres: 0.58μm and 1.04μm in diameter. Mie scattering calculations^48^ at λ = 633 nm with microsphere refractive index = 1.59 and that for water = 1.33 yield: for the 1.04μm microspheres a scattering efficiency Q = 2.68, an anisotropy factor g = 0.92 (average scattering angle of 23°); for the 0.58μm microspheres: scattering efficiency Q = 1.01, g = 0.85 (average scattering angle of 32°). The associated concentrations of the microsphere suspensions were 0.004 spheres/μm^3^ of 1.04-μm-diam spheres for a scattering coefficient of 100 cm^−1^, and 0.038 spheres/μm^3^ of 0.58-μm-diam spheres respectively. For other increasing scattering coefficient samples, the sphere concentration was adjusted accordingly.

#### Experimental setup

The speckle images were generated by illuminating the optical phantoms (2 × 2 × 2cm^3^) with a HeNe laser at a wavelength of 632.8 nm emitting 19 mW and with a coherence length of 20–30 cm. The laser power was measured to be 11 mW incident on the phantom surface. The long coherence length is appropriate so that interference after multiple scattering still creates speckle patterns. A half-wave plate and a linear polarizer controlled the input light polarization to be vertical (perpendicular to the table). The backscattered speckle pattern passed through an objective lens and was captured with a CCD camera (Lucid, Triton 5MP Polarization Model) at an off-axis angle of 30° at a distance of 23 cm (see Figure S1). The camera has a standardized Type 2/3″ sensor (11.1 mm diagonal) containing 2048x2448 pixels, resolving the image at a pixel size of 3.45 μm × 3.45 μm. For statistical purposes, ten speckle images were acquired per sample. Typical speckles detected in this study comprised of ∼10–20 image pixels, safely satisfying the Nyquist sampling criteria and ensuring that the calculations of speckle size and contrast are independent of each other.^35^^,^^49^ To avoid blurring of speckles due to Brownian motion of scatterers,^21^^,^^23^^,^^50^ which would increase speckle size and decrease contrast, a short (sub-millisecond) exposure time was used throughout the study.

#### Monte Carlo simulation

The Monte Carlo (MC) simulations used here to help interpret experimental findings and provide insights into volumetric speckle patterns were based on a validated polarization-sensitive Monte Carlo code developed by our group.^44^^,^^51^^,^^52^^,^^53^^,^^54^^,^^55^^,^^56^^,^^57^^,^^58^^,^^59^^,^^60^ This is an intensity-based simulation that doesn’t render speckle patterns, but is used here to estimate the subsurface fluence pattern statistics of the photons that propagated through the sample and emerged through the illuminated surface. Specifically, we tabulated the FWHM diameter of the backwards-emitted surface intensity (to correspond to D_l_ in Equation 1), the total average photon path length and average number of scattering events of the detected photons. The parameters chosen for the simulations mimicked the experimental conditions. The input light was chosen to be collimated beam 2 mm in diameter (top-hat profile). The scattering properties were based on spherical Mie scatterers (the diameter and concentration were changed in accordance with experiments). The short experimental exposure time permits treating the scattering sample as a static object when running the Monte Carlo simulations. Typical photon numbers per simulation were ∼10^7^, and each simulation was repeated 5 times to improve precision and robustness. Variations in the estimated photon statistics are estimated by the standard deviation of the 5 repetitions.

### Quantification and statistical analysis

#### Speckle size and contrast

To quantify the stochastic properties of the acquired speckle patterns, we calculated two independent metrics: (1) speckle size and (2) speckle contrast. Speckle size is a one-dimensional measure of the average spatial correlation area of the intensity in a speckle pattern. It is conveniently determined by averaging the FWHM of the autocorrelation of each row along one dimension of the image (see Data S1). Considering the slight off-axis setup along the horizontal direction, the speckle size reported in this paper is the vertical average correlation area. It is theoretically predicted to depend on sample properties and measurement geometry according to^61^^,^^62^

(Equation 1) $$d_{y}=1.22\;\frac{\lambda *D}{D_{I}*\cos\left(\theta \right)}$$

where d_y_ represents the average speckle size (what we refer to as ‘effective diameter’ in this paper), D the distance between sample and camera, D_I_ is the linear dimension of the light spot exiting the turbid sample after multiple scattering events, θ the off-axis measurement angle and λ the wavelength of the light. As wavelength, distance between sample and camera, and the measurement angle are constant, the speckle size dependence on medium properties is only through the D_l_ term above, or the spatial extent of the emitting region on the surface of the illuminated sample. The inversely proportional relationship between speckle size and the extent of the light emitting region on the sample aligns with the Van Cittert Zernike theorem^25^ which posits that a larger spread of light leads to a narrower autocorrelation function and thus smaller speckle size. As delineated by Goodman et al.,^25^ measuring outside the imaging (focal) plane of the objective is equivalent to measuring speckles in free space geometry. Speckle size trends will then primarily depend on the spread of light D_I_ and not on the numerical aperture of the lens, as would be the case when speckles are measured in imaging geometry. And while D_I_ will depend on the details of light propagation within the medium and thus its scattering properties, clearly additional speckle metrics are needed for improved and unambiguous sample characterization.

Toward that goal, the contrast metric of a speckle pattern was assessed by the ratio of intensity fluctuations, measured by the standard deviation std(I), to **mean** intensity ⟨I⟩ across the whole image

(Equation 2) $$C=\frac{std\left(I\right)}{\left\langle I\right\rangle }$$

While theories for speckle generated from rough surfaces exist,^25^^,^^30^^,^^63^ relationships between volumetric material properties and resultant speckle patterns are lacking. General considerations posit, for example, that a medium with fewer but stronger scatterers (larger scattering cross-section per scatterer) will yield higher speckle contrast than a medium with more but weaker scatterers.^64^ Our study will in part test these dependencies, and in part lay a more solid foundation for relating volumetric medium scattering properties to resultant speckle patterns.

For statistical purposes, results as shown in Figures 1 and 2 are obtained by analyzing 10 consecutive speckle images per sample and average speckle size and contrast measurements. This number was reduced for tissue imaging, acquiring 5 speckle patterns in healthy and 5 in tumor tissue. The errors of the average speckle size and contrast are estimated by the standard deviation. As speckle patterns from phantoms and tissue are acquired in sub-millisecond time frames that are shorter than dynamic processes occurring in the samples, both can be treated as static snapshots of the system and any ergodicity differences estimated via the standard deviation of multiple measurements.

## Supplemental information

Document S1. Figure S1

Data S1. PDF file containing MATLAB code for calculating the speckle size and speckle contrast of the speckle pattern, related to STAR Methods
