# Supplementary material for: Laser speckle size and contrast investigation of volumetric scattering from controlled turbid phantoms and mouse skin tissues
Source: iScience. 2025 Apr 15;28(5):112433. doi: 10.1016/j.isci.2025.112433 (PMC12063137; doi:10.1016/j.isci.2025.112433)
Supplement: Document S1. Figure S1 [file mmc1.pdf]

iScience, Volume *28*

Supplemental information

Laser speckle size and contrast investigation of volumetric scattering from controlled turbid phantoms and mouse skin tissues

Carla Kulcsar, Daniel C. Louie, and Alex Vitkin

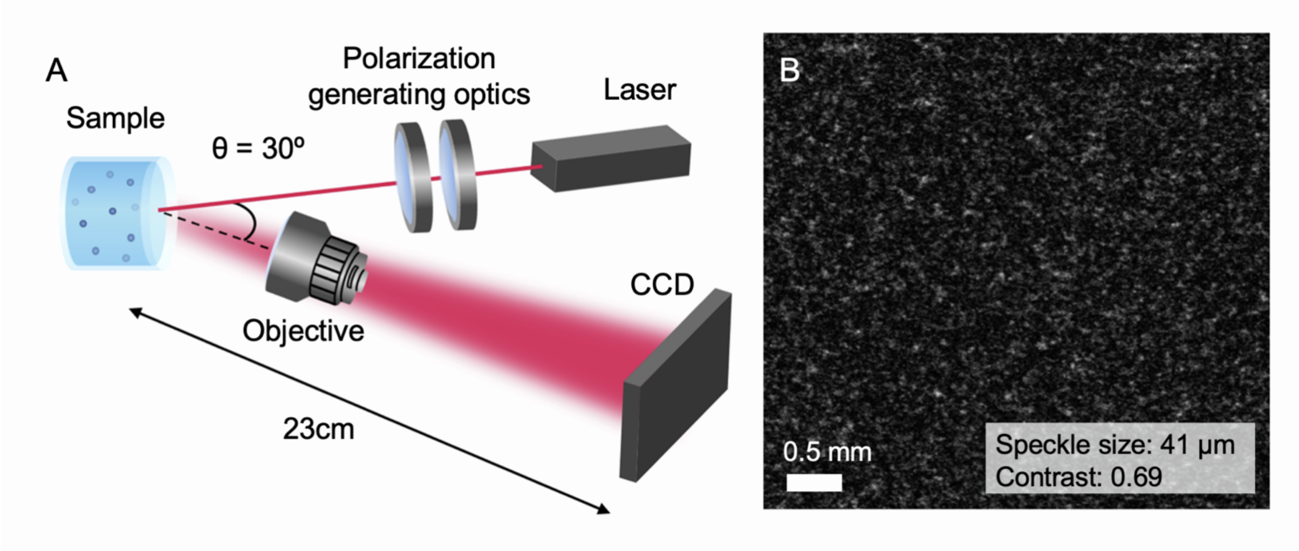

Figure S1 Experimental setup and illustrative speckle pattern, related to STAR Methods: A) Experimental setup for speckle measurement of microsphere suspensions. Coherent light from the HeNe laser passes through polarization optics (half-wave plate and linear polarizer). The polarized light beam illuminates the optical phantom at normal incidence. The backscattered speckle pattern is magnified by an objective lens and is captured by a CCD camera. **B)** Illustrative speckle pattern acquired from a 1.04-μm-diam microsphere suspension with a scattering coefficient of 300 cm^{-1}. The scalebar represents 0.5 mm.
