# Supplementary material for: Laser speckle size and contrast investigation of volumetric scattering from controlled turbid phantoms and mouse skin tissues
Source: iScience. 2025 Apr 15;28(5):112433. doi: 10.1016/j.isci.2025.112433 (PMC12063137; doi:10.1016/j.isci.2025.112433)
Supplement: Data S1. PDF file containing MATLAB code for calculating the speckle size and speckle contrast of the speckle pattern, related to STAR Methods [file mmc2.pdf]

Data S1 Matlab code for calculating the speckle size and speckle contrast of the speckle pattern, related to STAR Methods

```
% SPECKLESIZE
% image = speckle pattern image, 2048x2448 pixel speckle pattern image from CCD camera

crosscorr_sum = zeros(2*size(image, 1)-1, 1);

for w = 1:(size(image, 2)) % calculate autocorrelation function for every line in the image
    crosscorr = xcov(image(:, w), 'normalized');
    crosscorr_sum = crosscorr_sum + crosscorr;
end

crosscorr_average = crosscorr_sum/(size(image, 2)); % take the average autocorrelation

% use the matlab 'find' index function (find(crosscorr_average >= 0.5, 1, 'first')) to find the half maximum
of the autocorrelation function and interpolate to obtain the Full-width-half-maximum of average
autocorrelation function in pixel %


% CONTRAST
C = std(image(:))/mean(image(:)); %standard deviation devided by mean intensity of speckle image
```
